# Moderate drop in water table increases peatland vulnerability to post-fire regime shift

**DOI:** 10.1038/srep08063

**Published:** 2015-01-27

**Authors:** N. Kettridge, M. R. Turetsky, J. H. Sherwood, D. K. Thompson, C. A. Miller, B. W. Benscoter, M. D. Flannigan, B. M. Wotton, J. M. Waddington

**Affiliations:** 1 School of Geography, Earth and Environmental Sciences, University of Birmingham, Edgbaston, Birmingham, B15 2TT, UK; 2 Department of Integrative Biology, University of Guelph, Guelph, Ontario, N1G 2W1, Canada; 3 School of Geography and Earth Sciences, McMaster University, Hamilton, Ontario, L8S 4K1, Canada; 4 Natural Resources Canada, Canadian Forest Service, Northern Forestry Centre, Edmonton, Alberta, T6H 3S5, Canada; 5 Department of Biological Sciences, Florida Atlantic University, Davie, Florida 33314, USA; 6 Department of Renewable Resources, University of Alberta, Edmonton, Alberta, T6G 2H1, Canada; 7 Faculty of Forestry, University of Toronto, Toronto, Ontario, M5S 3B3, Canada; 8 Natural Resources Canada, Canadian Forest Service, Great Lakes Forestry Centre, Sault Ste Marie, Ontario, P6A ZES, Canada

## Abstract

Northern and tropical peatlands represent a globally significant carbon reserve accumulated over thousands of years of waterlogged conditions. It is unclear whether moderate drying predicted for northern peatlands will stimulate burning and carbon losses as has occurred in their smaller tropical counterparts where the carbon legacy has been destabilized due to severe drainage and deep peat fires. Capitalizing on a unique long-term experiment, we quantify the post-wildfire recovery of a northern peatland subjected to decadal drainage. We show that the moderate drop in water table position predicted for most northern regions triggers a shift in vegetation composition previously observed within only severely disturbed tropical peatlands. The combined impact of moderate drainage followed by wildfire converted the low productivity, moss-dominated peatland to a non-carbon accumulating shrub-grass ecosystem. This new ecosystem is likely to experience a low intensity, high frequency wildfire regime, which will further deplete the legacy of stored peat carbon.

Wildfire has been an important disturbance within the boreal region of North America throughout the Holocene^1^. Numerous studies demonstrate that northern peatlands are resilient to this large scale disturbance^2^. The ecophysiological traits of *Sphagnum* mosses that carpet most peatland surfaces lead to an array of negative feedbacks^3^ that have enabled peatlands to persist for millennia. The high specific yield of peat (the addition of water required to induce a unit rise in the water table position^4^), limits fluctuations in the water table position, maintaining near saturated conditions. Further, small leaved *Sphagnum* species that dominate high latitude peatlands resist all but the most intense wildfires by retaining high near-surface moisture contents^5^. These traits protect the peatland carbon stock from deep peat combustion and allows for the rapid re-establishment of ecosystem function and the return to a net carbon storage within several years post-fire^6^.

Major hydrological disruption in tropical peatlands has resulted in catastrophic wildfires and the loss of peatland ecosystems. In 1997, wildfires in drained Indonesian peatlands led to the release of 0.95–2.57 Gt of carbon into the atmosphere^7^,^8^. While peatlands at high latitudes are not in general subject to the intensive drainage occurring in tropical regions, projected increases in evapotranspiration with climate change will likely exceed precipitation increases across the boreal region, leading to drying of these northern peatlands^9^. The boreal wildfire regime is also shifting in conjunction with this climate-induced drying. The annual area burned across boreal North America doubled between 1960 and 1990^10^ and is projected to increase by up to a further 118% by 2100^11^ if wildfire is not limited by fuel availability^12^. The vulnerability of northern peatlands to these changes is unknown, yet it is critical for predicting the fate of northern high latitude carbon stocks.

To investigate the fate of high latitude carbon stocks following drainage and wildfire, we capitalized on a unique long-term experiment. A treed fen in Alberta, Canada was partially drained for silviculture in 1983. The water table position was immediately lowered by about 0.25 m, comparable to projections of future water table reductions (ranging from 0.05 to 0.6 m)^9^. The hydrological impact diminished over time as trenches used for drainage infilled, resulting in a 0.10 m difference in water table position between drained and undrained portions of the fen in 2003. In 2001, a 105,000 ha wildfire burned both the drained and undrained portions of the fen^13^.

## Results

**Figure 1:**
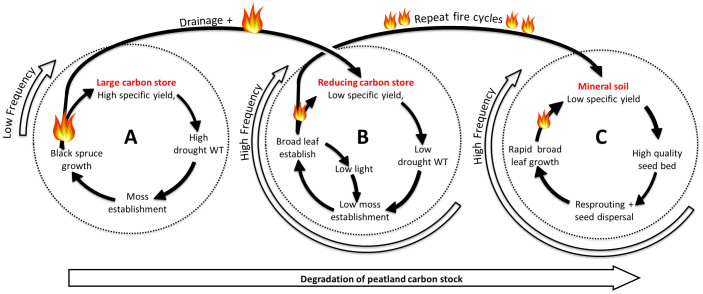
Conceptual diagram showing the degradation of the peatland carbon stock. Far left cycle (A) represents the low frequency peatland fire cycle. The compound disturbance of fire and drainage breaks this cycle, transferring the peatland to the central fire cycle (B). This represents a spiral of decline in peatland carbon stocks. After repeated high frequency fire cycles, peatland stocks are lost to the atmosphere and the landscape is transferred to a stable mineral soil fire cycle (C).

**Figure 2:**
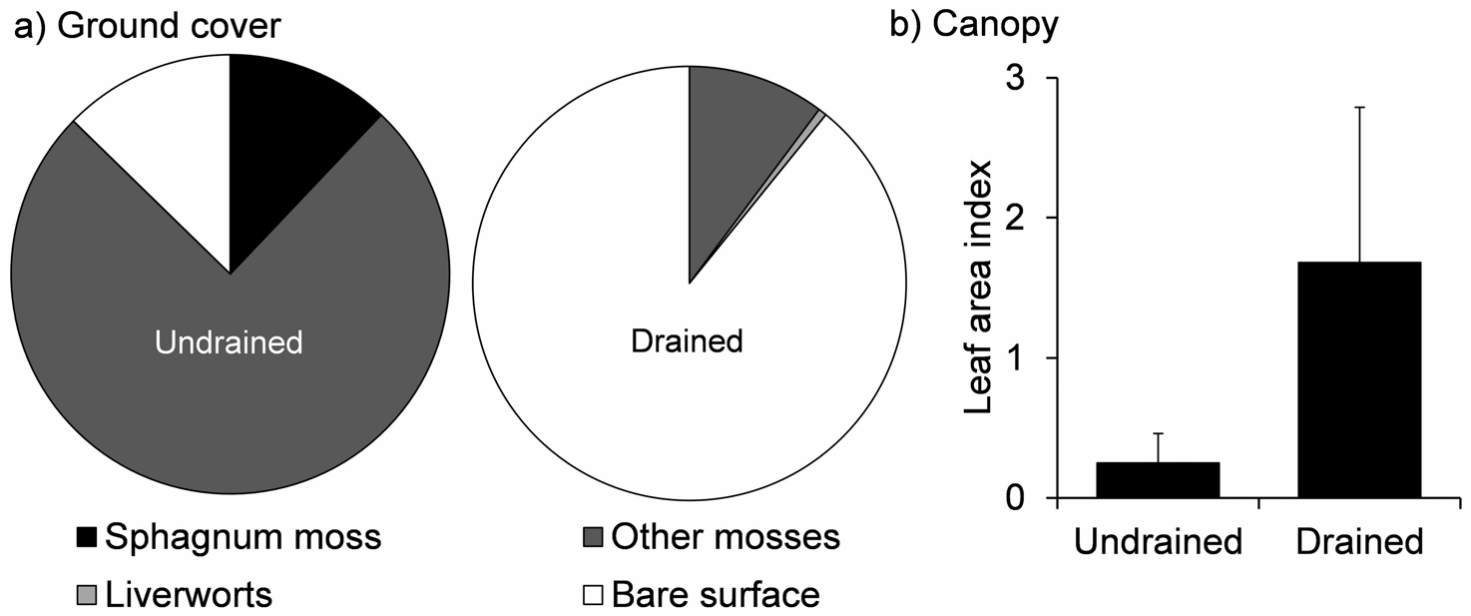
The compound effects of drying and fire on vegetation recovery. Aggregated percentage ground cover (a) and canopy leaf area index (b) within the undrained and drained plots 10 years post fire. The mature canopy of the drained site was comprised of seven different Salix (willow) species and two different types of Betula (birch). Within the undrained site, only young saplings of bog willow and birch were identifiable. See Table S1 for more detailed vegetation characterisation of ground cover.

In the absence of drainage, the peatland followed a typical pattern of post-fire recovery^6^,^14^ (Figure 1; cycle A). After wildfire, the specific yield remained high (0.59 within the top 0.1 m of the profile)^14^, maintaining a high water table position (maximum water table depth of 0.13 m during 2010 growing season). This likely promoted the observed establishment of a moss-dominated ground layer within a decade since fire (Figure 2a). This reestablishment of the peatland ecosystems results in a net accumulation of carbon 10 years post disturbance^6^ which will begin to offset carbon lost during the wildfire. This stable pattern of vegetation succession promotes peat accumulation and is exemplifying the resilience of peatlands to fire.

Drainage altered the recovery of vegetation following fire. There was only an 8.8% similarity (Sørensen's quotient) in vegetation between drained and undrained plots ten years post-fire. We observed the absence of *Sphagnum* moss cover at the drained site and a 77% reduction in total moss cover (Figure 2a). This was concurrent with a lower maximum water table depth (drained 0.36 m; undrained 0.13 m) and higher maximum peat tension (drained 0.56 m; undrained 0.25 m) during the 2010 growing season. The drained site was instead colonized by a mature canopy of willow and birch. The leaf area index of this canopy was significantly higher than the undrained site (p<0.001, t = 6.2; Figure 2b). This significantly reduced the availability of light at the ground surface compared to the undrained site (p <0.001; t = 6.9); total light transmissivity, drained site = 43.6 ± 27.4%, undrained site plots = 85.4 ± 11.9% (mean ± st dev).

## Discussion

**Figure 3:**
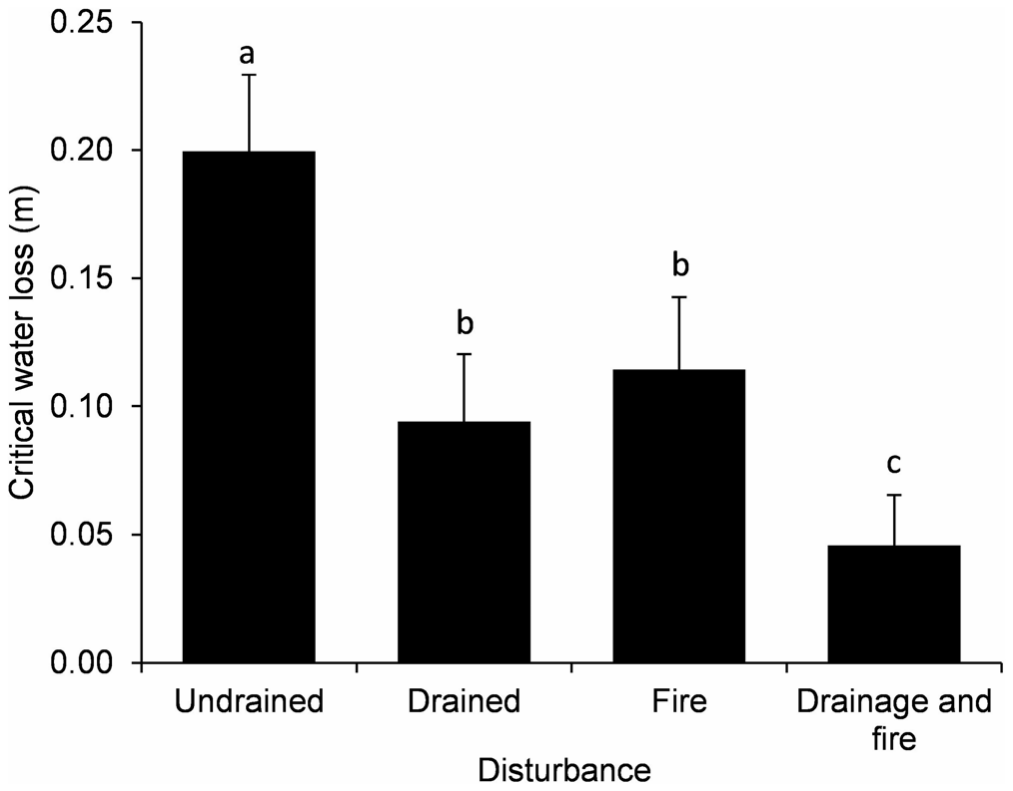
Loss of water required to lower the water table position within peatlands subject to different disturbances from the surface to a critical depth of 0.4 m; an ecohydrological threshold above which *Sphagnum* reestablishment is inhibited^16^,^17^. Values derived from analysis of peat hydrophysical properties measured before^26^ and after^15^ the wildfire within the drained and undrained sections of the peatland. Bars with same letter are not significantly different (p>0.05).

A modification of the peatland hydrological function likely instigated the observed shift in vegetation recovery and associated carbon dynamics observed at this unique experimental site. Drainage and fire each reduced the size of the critical hydrological buffer that regulated the near-surface moisture content and maintained the low tensions necessary for *Sphagnum* recolonisation^15^. Within the undisturbed peatland, 0.2 m of water loss via evapotranspiration and runoff was required to lower the water table depth by 0.4 m and trigger *Sphagnum* stress (critical water loss; Figure 3). Prior to fire, drainage compacted the peat profile, reducing this critical water loss by 51% compared to the undrained peatland (Figure 3; p<0.001, t = 7.0), while wildfire in the absence of drainage combusted poorly decomposed near-surface peat, reducing the critical water loss by 41% (Figure 3; p<0.001, t = 5.9). Together, the additive effects of drainage and wildfire reduced the critical water loss by 73% of that within the undisturbed peatland (pre-drainage and pre-fire; Figure 3; p<0.001, t = 11.6). This low critical water loss left the peatland vulnerable to high surface tension under drought conditions that likely limited *Sphagnum* establishment^16^,^17^ and promoted the observed invasion by broad leaf vegetation. The transition to a deciduous canopy also provided additional leaf litter^18^ and low light transmissivities^19^ that have been shown to restrict the establishment of moss species, reinforcing the observed change from a peatland to a shrub ecosystem.

Based on understanding gained from this *in situ* experiment, we propose a new conceptual model that projects the future degradation of peat carbon stores under drained and burned scenarios (Figure 1). Within this conceptual model, the combined impact of drainage and wildfire has the potential to transform a drained peatland with a long fire return interval to a shrubland with higher fire frequency (Figure 1; cycle B). We suggest that the rapid growth of the deciduous shrub canopy in the drained scenario can promote a high frequency, low intensity fire cycle^20^ in which the compact, high density peat^15^ is vulnerable to deep smouldering^21^. Under these circumstances, it seems likely that net carbon losses will occur across successive fire cycles, potentially leading to a progressive decline in the peatland carbon stock and the eventual formation of a stable^22^ upland ecosystem with a small carbon store associated with the current vegetation community^20^ (Figure 1; cycle C). Eventual exposure of mineral soils would provide a high quality seed bed, relative to organic soils, which would promote the establishment and growth of broad lead leaf vegetation^23^ after fire. We suggest that all of these changes have the potential to contribute to higher frequency fires, preventing the accumulation of organic matter^24^ and promoting a state change away from a peatland ecosystem^23^. Though our results are limited to our particular study, we believe that this conceptual model offers new opportunities to explore the ecohydrological and biogeochemical function of peatlands within a disturbance and resilience framework.

Overall, the results of this unique experiment suggest that severe drainage, such as that observed in tropical regions, may not be required to induce state changes in high latitude peatlands. Given that water table positions within peatlands across the boreal region are projected to drop by the magnitude observed within the drained site^9^, and that wildfire extent is expected to double by the end of the 21^st^ century^11^, findings from our study site highlight a potentially important future shift in peatland ecosystem form and function in response to changing disturbance regimes, and suggest that the peatland carbon stock could become more vulnerable to loss if climate- or human- induced drying modifies feedbacks between peatland vegetation and hydrology^3^.

## Methods

### Description of study site

Research was conducted at the Saulteaux River poor fen, 37 km south-east of the town of Slave Lake, Alberta, Canada (55.13°N, 114.25°W). The study site, with a maximum peat depth of 4 m, is characteristic of this broad class of fen system that dominates Canada's Boreal Plains. The response of the site to disturbance is thus suggestive of the general response of these peatland ecosystems. In 1983 a 50-ha portion of the poor fen was drained through the construction of 0.9 m deep ditches spaced 40 m apart. The drained and undrained portions of the fen subsequently burned in 2001 as part of a 105,000 ha wildfire (LWF-063)^25^. The fire consumed 0.19 ± 0.03 m of the surface peat in the drained portion of the peatland^13^ and 0.07 ± 0.01 m in the undrained portion. Further it resulted in complete tree mortality across the majority of drained and undrained areas.

### Hydrological measurements

Sampling transects established prior to the wildfire in the drained and undrained portions of the fen^26^ were reestablished during the 2010 growing season, nine years after the wildfire. Measurements were conducted parallel to previous transects to prevent interference (separation distance of 1 m). Within the drained site, the transect was aligned perpendicular to the ditch network. Ten groundwater wells (2 m in length; 50 mm i.d.) were installed at a spacing of 1 m. Water levels were continuously recorded at 15 minute intervals within the central well at each site, from day of year 150 to 240, using capacitance-based level recorder. Additional wells were manually measured every 7–10 days. Soil-water pressure was measured with 2.2 cm (o.d.) porous ceramic cup-type tensiometers at the drained and undrained sites, at 0.05, 0.15 and 0.30 m below surface, at the 0, 5 and 10 m points along the transects.

### Hydrological analysis

The loss of water required to lower the water table from the surface to a depth of 0.4 m within drained and undrained areas prior to fire were determined from specific yield measurements conducted by reference 14 and 26. Specific yield determines the volume of water loss per unit area necessary to lower the water table by a given depth. Prior to the wildfire, specific yield was measured at 0.10 m depth increments through nine peat cores along the transect within both the drained and undrained portions of the peatland. The water loss required to lower the water table to a depth of 0.4 m within the drained and undrained site was determined by averaging specific yield measurements over the top 0.4 m of the peat profile. The vertical variation in specific yield within the drained peatland after wildfire was determined by removing the top 0.19 m of the peat profile (that lost during the wildfire^12^) and extrapolating specific yield measurements to a depth of 0.4 m. Vertical variations in specific yield within the undrained peatland after fire was obtained from nine peat cores extracted from along the transect in 2010, within which specific yield was determined at 0.05 m increments. The significance of observed differences in the water loss necessary to lower the water table to a depth of 0.4 m between undrained, drained, fire and the combined impact of drainage and fire was established through t-tests.

### Species absence and mobility

Understory and surface plant species were identified within eight 0.5 m^2^ quadrats randomly distributed within the vicinity of each well transect. Sampling occurred during July 2010 while herbaceous and sedge species were flowering to ease species identification. Species presences within each quadrat was determined and percentage ground cover was also estimated for each quadrat. Species similarity between the drained and undrained portion of the peatland was determined using Sørenson's quotient of similarity (Q/S): 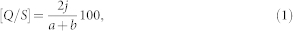
where *j* the number of species common to both samples, *a* is the total number recorded in the first sample, *b* is the number recorded in the second sample.

### Light transmissivity, canopy openness and leaf area index

Light transmissivity and leaf area index (LAI) were calculated using gap light photography. Hemispherical photographs were captured adjacent to the drained and undrained transects (n = 40) and within the center of the 16 species identification plots using a Nikon D60 camera and a Sunex 185° Super Fisheye lens. Photographs were processed using GLA v.2 software^27^ for total light transmissivity and leaf area index. Statistical differences in the canopy structure were determined through t-tests performed within SPSS.

## Author Contributions

N.K., M.R.T. and J.M.W. wrote the manuscript and devised the conceptual understanding. J.H.S., J.M.W., M.R.T., D.K.T. and B.W.B. devised the field research, J.H.S., D.K.T. and C.A.M. undertook the field research and J.H.S., N.K. and J.M.W. carried out the data analysis. M.D.F. and B.M.W. provided specific expertise and knowledge throughout the project. J.H.S., D.K.T., B.W.B., M.D.F., B.M.W. commented on the manuscript through its development. Funding was provided by M.R.T., B.M.W. and J.M.W.

## Supplementary Material

Supplementary Information Table S1
