# Supplementary material for: Moderate drop in water table increases peatland vulnerability to post-fire regime shift
Source: Sci Rep. 2015 Jan 27;5:8063. doi: 10.1038/srep08063 (PMC4306970; doi:10.1038/srep08063)
Supplement: Supplementary Information — Table S1 [file srep08063-s1.pdf]

Supplementary information: Moderate drop in water table increases peatland vulnerability to post-fire regime shift

N. Kettridge, M.R. Turetsky, J.H. Sherwood, D.K. Thompson, C.A. Miller, B.W. Benscoter, M.D. Flannigan, B.M. Wotton, J.M. Waddington

Table S1: The compound effects of drying and fire on vegetation recovery. Aggregated percentage of ground cover within the undrained and drained plots 10 years post fire.

Life form	Species name	% cover	
		Undrained	Drained
Moss (*Sphagnum*)	*Sphagnum angustifolium*	0.4	
	Sphagnum fuscum	10.6	
	Sphagnum magellanicum	1.0	
Moss (other)	*Aulacomnium palustre*	55.0	10.2
	Bryum pseudotriquetrum	3.1	
	Campylium stellatum	1.3	
	Drepanocladus fluitans	9.6	
	Helodium blandowii	1.0	
	Pleurozium schreberi	1.7	
	Tomenthypnum nitens	3.5	
Liverwort	*Marchantia polymorpha*		0.6
-	No live vegetation cover	12.7	89.2
